# Longitudinal Analysis of Natural Killer Cells in Dengue Virus-Infected Patients in Comparison to Chikungunya and Chikungunya/Dengue Virus-Infected Patients

**DOI:** 10.1371/journal.pntd.0004499

**Published:** 2016-03-03

**Authors:** Caroline Petitdemange, Nadia Wauquier, Hervé Devilliers, Hans Yssel, Illich Mombo, Mélanie Caron, Dieudonné Nkoghé, Patrice Debré, Eric Leroy, Vincent Vieillard

**Affiliations:** 1 Sorbonne Universités, UPMC Univ Paris 06, INSERM U1135, CNRS ERL8255, Centre d’Immunologie et des Maladies Infectieuses (CIMI-Paris), Paris, France; 2 Centre International de Recherches Médicales de Franceville (CIRMF), Franceville, Gabon; 3 Metabiota, Inc., San Francisco, California, United States of America; 4 Internal Medicine and Systemic Diseases Department, Dijon University Hospital, Dijon, France; Florida Gulf Coast University, UNITED STATES

## Abstract

**Background:**

Dengue virus (DENV) is the most prominent arbovirus worldwide, causing major epidemics in South-East Asia, South America and Africa. In 2010, a major DENV-2 outbreak occurred in Gabon with cases of patients co-infected with chikungunya virus (CHIKV). Although the innate immune response is thought to be of primordial importance in the development and outcome of arbovirus-associated pathologies, our knowledge of the role of natural killer (NK) cells during DENV-2 infection is in its *infancy*.

**Methodology:**

We performed the first extensive comparative longitudinal characterization of NK cells in patients infected by DENV-2, CHIKV or both viruses. Hierarchical clustering and principal component analyses were performed to discriminate between CHIKV and DENV-2 infected patients.

**Principal Findings:**

We observed that both activation and differentiation of NK cells are induced during the acute phase of infection by DENV-2 and CHIKV. Combinatorial analysis however, revealed that both arboviruses induced two different signatures of NK-cell responses, with CHIKV more associated with terminal differentiation, and DENV-2 with inhibitory KIRs. We show also that intracellular production of interferon-γ (IFN-γ) by NK cells is strongly stimulated in acute DENV-2 infection, compared to CHIKV.

**Conclusions/Significance:**

Although specific differences were observed between CHIKV and DENV-2 infections, the significant remodeling of NK cell populations observed here suggests their potential roles in the control of both infections.

## Author Summary

Dengue fever is the most important arthropod-borne viral disease worldwide, affecting 50 to 100 million individuals annually. The clinical picture associated with acute dengue virus (DENV) infections ranges from classical febrile illness to life-threatening disease. The innate immunity is the first line of defense in the control of viral replication. In this article, we examine the particular role of natural killer (NK) cells in DENV infection at different time points after the onset of symptoms. This extensive study was performed in comparison with patients infected by Chikungunya virus (CHIKV), another major arbovirus transmitted by the same mosquito vectors, and co-infected CHIKV/DENV-2 patients. We observed that DENV2 and CHIKV induced different signatures of NK-cell responses suggesting specific roles in the control of both infections.

## Introduction

Dengue virus (DENV), the most widespread arbovirus worldwide, is transmitted by *Ae*. *aegypti* and *Ae*. *albopictus* mosquitoes and is responsible for major outbreaks causing serious health and economical problems. Dengue is endemic in at least 100 countries in Southeast Asia, the pacific islands, the Americas, Africa, and the Caribbean and the World Health Organization (WHO) estimates that 50 to 100 million infections occur yearly [1,2]. Chikungunya virus (CHIKV), another arbovirus also transmitted by the mosquito vectors *Ae*. *aegypti* and *Ae*. *Albopictus*, reemerged prominently in 2004 [3]. The expanding geographical distribution of *Ae*. *albopictus* has led to an increase in overlap of DENV and CHIVK epidemic geographic regions and co-infections in humans have been reported [4,5]. During a large outbreak in 2010 in Gabon, both viruses were detected in a single mosquito caught in the wild, providing evidence of their potential simultaneous transmission to humans [4].

DENV and CHIKV infections cause acute illness characterized by a broad spectrum of shared clinical symptoms including high fever, myalgia, headache, joint point, skin rash and vomiting. Dengue fever (DF) is caused by any of four closely related viruses DENV 1–4 with a fifth serotype identified recently [6]. Primary infection with one serotype of DENV confers only short-term partial cross-protection against other serotypes. Sequential infections put patients at greater risk of developing dengue hemorrhagic fever (DHF) and dengue shock syndrome (DSS) [7]. However, interestingly, the risk of developing a severe form may be higher during a secondary infection compared to a third or a forth (post-secondary) [8]. Most clinical symptoms of DENV and CHIKV related-diseases resolve within a few weeks with the exception of CHIKV-associated joint pains that can persist for longer periods [3,9].

The innate immune response constitutes the first line of defense against pathogenic microorganisms, and is particularly important in the early control of viral infections [10]. Natural Killer (NK) cells are a key component of the innate immune defense, capable of recognizing and destroying target cells during early infectious events. A delicate balance of activating and inhibitory signals regulates the ability of NK cells to kill target cells and secrete cytokines, allowing them to distinguish between healthy and virus-infected cells. The main inhibitory receptors, including the killer cell immunoglobulin (Ig)-like receptors (KIR), CD94/NKG2A and ILT-2, recognize distinct histocompatibility complex (MHC) class I molecules [11]. It seems that a critical threshold of signaling via activating receptors exceeding the counterbalancing influence of inhibitory receptors must be reached in order for NK cells to mount a productive response [12]. These activating receptors include CD94/NKG2C, NKG2D, DNAM-1 and the natural cytotoxicity receptors (NCR); NKp30, NKp44 and NKp46 [11,13].

We recently provided evidence that CHIKV could shape the NK cell repertoire through a clonal expansion, of NKG2C^+^ cytototoxic NK cells, in correlation with the viral load [14]. These results suggested that NK cells are able to sense CHIKV early during the course of infection and may thus contribute to viral clearance. Other studies suggest that NK cells could also play a role in the response against DENV infection however the data is sparse. In summary, a higher absolute number of NK cells associated with cell-activation were reported in patients who developed acute DF [15–19], and in mouse model [20].

This study aimed to explore the repertoire of NK cells in DENV-2-infected patients, in comparison with CHIKV-infected patients and CHIKV/DENV-2 co-infected patients. Taken together, our results reveal a general expansion of highly activated and differentiated NK cells in DENV-2, CHIKV and CHIKV/DENV-2 infected patients, although some specific NK receptors were more strongly associated to DENV-2 or CHIKV. Furthermore, we observed the persistence of fully differentiated NKG2C^+^CD57^+^ NK cell in association with viral load in CHIKV^+^ convalescent patients only.

## Methods

### Ethical considerations

We used surveillance data collected by the Viral Emerging Diseases Unit (UMVE) at the International Center For Medical Research at Franceville (CIRMF), partnered with the Gabonese Ministry of Health and Sanitation (MoHS). From April to July 2010 a simultaneous outbreak of CHIKV and DENV-2 occurred in both Ogooue Lolo and Haut Ogooue provinces, in southeast Gabon, central Africa. Epidemiological and clinical inquiries as well as blood sampling for laboratory confirmation were considered as part of the public health response. In compliance with the Gabonese MoHS, consent was obtained for each patient during interviews. The Regional Health Director approved the study and the consenting strategy (Authorization n°189). All investigations were conducted in compliance with the principles for medical research involving human subjects expressed in the Declaration of Helsinki.

### Patients, healthy controls and sample preparation

In the case definition adopted by the Gabonese MoHS, an acute febrile illness was characterized by acute fever (>38.5°C), and >1 of the following symptoms: arthralgia, myalgia, headache, rash, fatigue, nausea, vomiting, diarrhea or bleeding. We excluded patients whose symptoms met these criteria but who had laboratory-confirmed malaria. Patients who met the suspect case definition were sampled and tested for virological and cellular investigations. The kinetics study used early acute samples collected between days 0–3, late acute samples collected between days 5–15 and convalescent samples collected at days>30, after the onset of symptoms. Isolation of peripheral blood mononuclear cells (PBMC) was performed by standard histopaque density centrifugation. Sex- and age-matched healthy volunteers from Franceville (Gabon) were used as controls [14]. The characteristics of patients and controls are summarized in Table 1. Patients and controls were negative for yellow fever, West Nile fever, Rift Valley fever, and malaria, as previously described [14].

**Table 1:** Characteristics of healthy donors and patients.

**Characteristics**	**NI**	**CHIKV**	**DENV-2**	**CHIKV/DENV-2**
Mean age in year	32	35	31	42
Male:Female (n)	6:9	12:8	11:7	4:5
Median CHIKV load	<10	5.3x10^4^	<10	5.0x10^4^
(cDNA/mL)		(<10–1.5x10^8^)		(<10–4.8x10^6^)
Median DENV load	<10	<10	3.1x10^7^	2.6x10^5^
(cDNA/mL)			(2.8x10^3^-2.4x10^8^)	(1.0x10^4^-9.4x10^7^)
Severe form of disease (%)	N/A	40.0	50.0	22.2
Arthralgia (%)	0	82.3	0	75.0

Abbreviations: NI, Non-infected Gabonese healthy donors; CHIKV, Patients infected by Chikungunya virus; DENV-2, Patients infected by Dengue virus type 2; CHIKV/DENV, Patients co-infected by CHIKV and DENV; cDNA, complementary DNA; Severe form of disease: patients with fever (>38.5°C) and more than one of the following symptoms: arthralgia, myalgia, headaches, rash, fatigue, nausea, vomiting, diarrhea or bleeding. N/A, Not applicable.

### Virological characterization

RNA was extracted from 140 μL of plasma using the QIAamp Viral RNA Mini kit (Qiagen). cDNA was synthesized using qRT-PCR with a 9700 thermocycler (Applied biosystems), and mixing 25 μL of extracted RNA with 25 μL of High Capacity cDNA kit (Applied Biosystems). Finally, 5 μL of newly synthesized cDNA was used as template in 25 μL of Taqman universal PCR Master Mix with specific CHIKV or DENV primers and run in a 7500 Real-time PCR system (Applied Biosystem), as described [4].

IgG seropositivy against Human Cytomegalovirus (HCMV) was assessed in plasma using the CMV IgG ELISA Kit from Sigma-Aldrich abiding to manufacturer’s recommendations.

### Flow cytometry analysis

PBMC were stained using the appropriate cocktail of antibodies, as described [14]: anti-CD45-KO (J33), anti-CD3-ECD (UCHT1), anti-CD56-PC7 (N901), anti-NKG2A-APC (Z199), anti-CD57-PB (NC1), anti-NKp44-PE (Z231), anti-CD16-PB (3G8), anti-ILT-2-PE (HP-F1), from Beckman coulter; anti-KIR2DL2/3-FITC (CH-L), anti-CD69-APC-Cy7 (FN50), anti-CD161-FITC (DX12), anti-HLA-DR-Alexa Fluor 700 (G46-6) from Becton Dickinson; anti-NKp30-APC (AF29-4D12) from Miltenyi Biotec; anti-NKG2C-PE (134591) from R&D Systems. Isotype-matched immunoglobulins served as negative controls. Cells were gated on the CD45^+^ lymphocytes gate. At least 20,000 CD45^+^ cells were analyzed on a Gallios cytometer (Beckman coulter). Flow cytometry data was analyzed using FlowJo software version 9.

### Cytolytic markers and functional assays

For intracellular staining of cytolytic enzymes, PBMC were fixed and permeabilized with a cytofix/cytoperm kit (Becton Dickinson) and stained with perforin-PE (δG9), or granzyme-B-FITC (GB1). To stimulate intracellular IFN-γ production, PBMC were incubated overnight in the presence of IL-12 (10 ng/mL) and IL-18 (100 ng/mL) (R&D Systems), prior to fixation/permeabilization, and stained with anti-IFN-γ mAb (B27; Becton Dickinson). Isotype-matched immunoglobulins served as negative controls as previously described [14].

Degranulation activity was assessed through the detection of surface marker LAMP1/CD107a on PBMC stimulated with HLA-class-I negative K562 target cells. Briefly, non-activated PBMC were resuspended in the presence of anti-CD107a mAb (H4A3, Becton Dickinson) with target cells at an effector:target (E:T) cell ratio of 1:1. After 1 h of incubation, monensin (Sigma Aldrich) was added at 2 mM for an additional 4 h of incubation [14].

Fluorescence was acquired with a Gallios cytometer (Beckman coulter). Flow cytometry data was analyzed using FlowJo software version 9.

### Statistical analysis

All statistical analyses were performed using Prism-5 software (GraphPad Software). Mann-Whitney tests were performed for individual comparisons of two independent groups, and the nonparametric Kruskal–Wallis test with Dunn post-test was used to define the significance of results from more than two independent groups of subjects, when compared 2 by 2. Nonparametric correlations were assessed by determination of Spearman rank correlation coefficient. P-values <0.05 were considered significant. *p<0.05; **p<0.01; ***p<0.001. A principal component analysis (PCA) was conducted to identify the most prevalent grouping cell-surface markers on CD3^-^CD56^+^ NK cells in the different samples’ groups (controls, DENV-2 and CHIKV). Data were represented on the correlation circle as a supplementary variable.

## Results

### Expansion of a highly activated and differentiated NK cell subset in DENV-2^+^ patients

**Fig 1:**
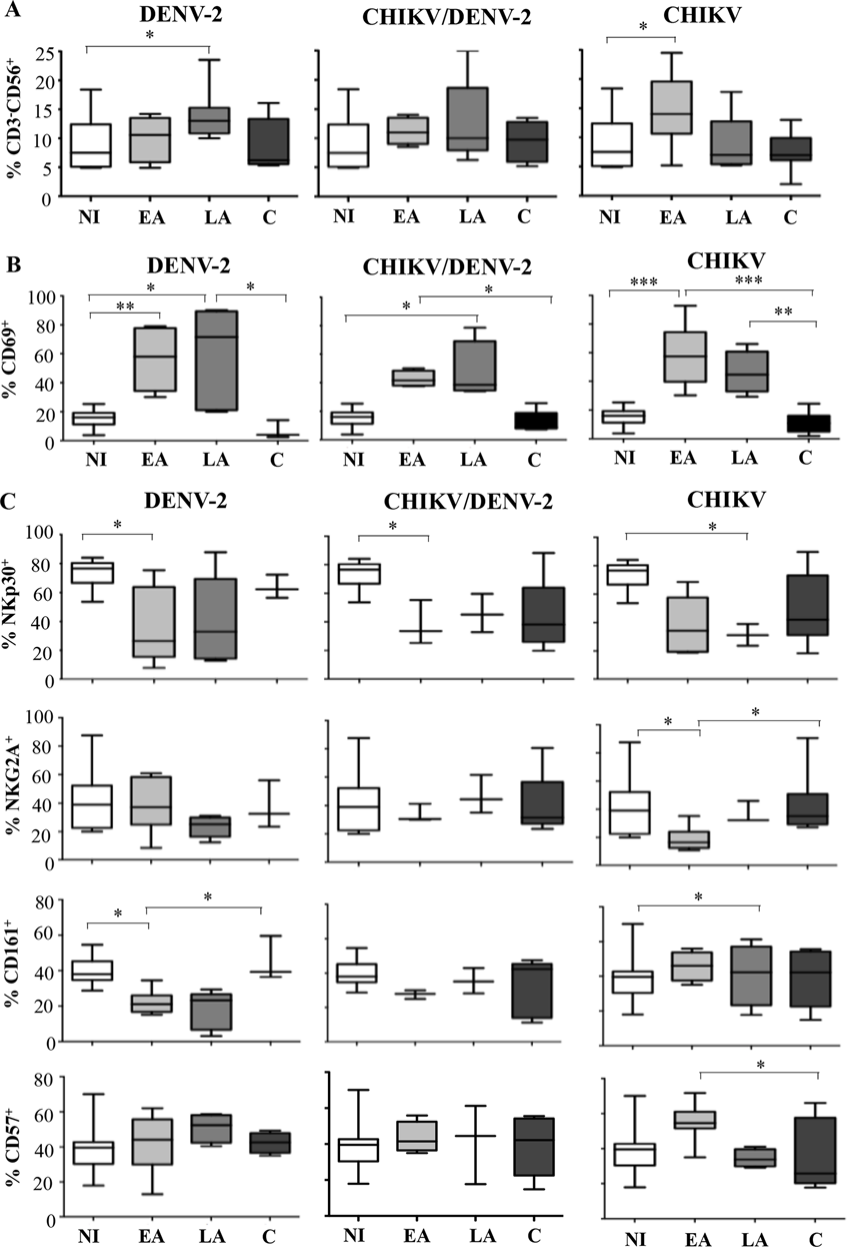
Frequencies and characteristics of NK cells in patients infected with DENV-2, CHIKV, co-infected with CHIKV and DENV-2 (CHIKV/DENV-2), and non-infected and healthy Gabonese controls (NI). Patients were sampled in early acute (EA; day (D)0-D3), late acute (LA; D12-D15) and convalescent (C; D>30) stages post-onset of symptoms. Frequencies of CD3^-^CD56^+^ NK cells within the CD45^+^ lymphocyte gate (A), frequencies of early activation marker CD69 (B) and NKp30, NKG2A, CD161, CD57 cell-surface receptors (C) on CD3^-^CD56^+^ NK cells are represented. * p<0.05, ** p<0.001.

Using multi-color flow cytometry, we performed an extensive characterization of NK cells in peripheral blood cells collected from DENV-2 infected patients, and compared the data to results obtained from CHIKV infected patients, CHIKV/DENV-2 co-infected patients, and healthy Gabonese individuals. As observed previously, NK cell frequency increased early after both DENV-2 and CHIKV infections but more rapidly in CHIKV-infected (Fig 1A) [14]. NK-cell frequencies gradually increased and peaked within days 5–15 after the onset of symptoms in DENV-2 mono-infected patients as well as in co-infected patients, versus 0–3 days in CHIKV infected patients (Table 1).

At the peak of infection, the proportion of NK cells expressing the early activation marker CD69^+^ was significantly increased in all patients and reached 72±35% in DENV-2, 58±21% in CHIKV^+^ and 42±5% in CHIKV/DENV-2 co-infected patients, as compared to 15±6% in healthy Gabonese controls (Fig 1B) [14,17]. This was also in accordance with early activation of NK cells in response to primary DENV infection in mice [20]. Activation of NK cells was also confirmed through the increased expression of HLA-DR and NKp44, two markers expressed on late-activated NK cells (S1 Fig). Importantly, one month after disease onset, the frequency of these activation markers decreased to match baseline levels observed in healthy individuals (Fig 1B and S1 Fig).

We set out to study the modulation of specific NK cell receptors in the different groups of patients. Compared to healthy donors, DENV-2^+^ patients’ NK cells expressed significantly less NKp30, NKG2A and CD161 (Fig 1C), whereas expression of ILT-2 was increased (S1 Fig). CHIKV^+^ and co-infected patients seemed to be characterized by a prolonged phenotypic modulation of NK cells. In all patient groups, NK cells were mainly CD56^dim^ (data not shown), as previously observed in a similar cohort of CHIKV-infected patients [14]. Taken together, these data suggest that both activation and differentiation of NK cells are induced during the acute phase of infection.

### Transient expression of NKG2C^+^CD57^+^ NK cells in DENV-2^+^ patients

**Fig 2:**
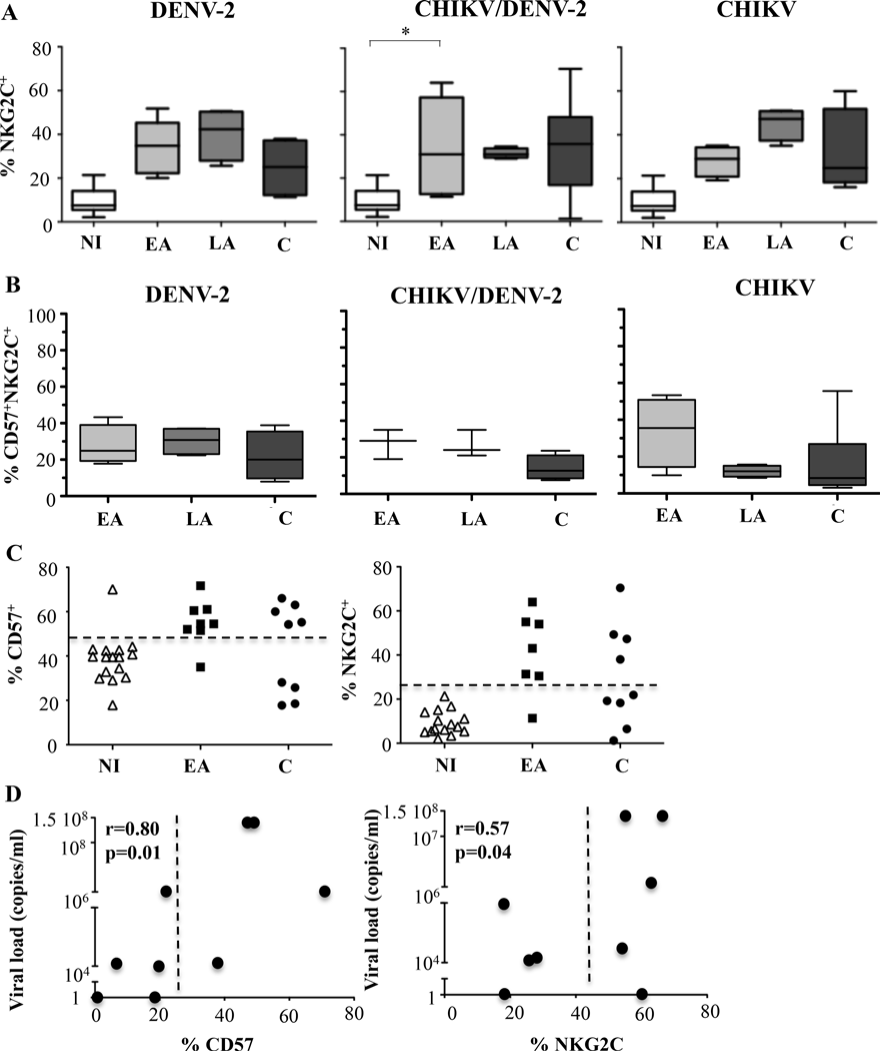
Persistence of NKG2C^+^CD57^+^ NK cells in CHIKV-infected patients with high viral loads. Results are grouped according to 1) type of infection: DENV-2, CHIKV or co-infected by CHIKV and DENV-2 (CHIKV/DENV-2), and non-infected Gabonese healthy controls (NI) and 2) date of sample collection after onset of symptoms: early acute (EA; day (D)0-D3), late acute (LA; D12-D15) and convalescent (C; D>30). (A) Frequency of NKG2C^+^ cells within CD3^-^CD56^+^ NK cells amongst all patient groups (B) Frequency of CD57^+^ NK cells within NKG2C^+^ or NKG2C^-^ NK cell-subsets from CHIKV-, DENV-2- and co-infected patients analyzed at Day 30 post-symptoms. (C) Scatter plot representation of CD57^+^ and NKG2C^+^ NK cell frequencies collected in CHIKV-infected patients. Dotted vertical lines represent the baseline levels. (D) Correlation between the set point of the viral load quantified during the early acute CHIKV infection and CD57^+^ or NKG2C^+^ NK cells analyzed at Day 30 post-symptoms. Dotted horizontal lines represent baseline levels. * p<0.05, ** p<0.001.

CHIKV and DENV-2 infections were both associated with a rapid and significant increase in the frequency of NK cells expressing NKG2C (Fig 2A), as previously described in CHIKV [13] and other viral infections, in association with CD57 [21–24]. Fig 2B shows that NKG2C^+^CD57^+^ NK cells increase in frequency and reach peak values at a different time points depending on the nature of the infection: at the early acute phase in CHIKV-infected patients and at the late acute phase in DENV-2-infected patients. However, this increase in frequency of CD57^+^ and NKG2C^+^ NK cells was transient, whatever the group of patients (Fig 2B). Of note, all different groups of patients presented very high HCMV seroprevalence rates; 87.5% IgG-HCMV^+^ in healthy controls, 89.5% in CHIKV^+^, 91.7% in DENV-2^+^ and 100% in co-infected patients. As expected, all HCMV negative patients expressed very low levels of NKG2C. However, following CHIKV infection, a subset of these highly differentiated NK cells persisted in some convalescent patients, more than 30 days after infection (Fig 2C). The proportion of persisting CD57^+^ and NKG2C^+^ NK cells correlated with the viral load quantified during the early acute CHIKV infection (Spearman coefficient: r = 0.57; p = 0.04 for CD57, and r = 0.80; p = 0.01 for NKG2C expression) (Fig 2D). This suggests that the viremia is only associated with the persistence of a specific, highly differentiated NK subset in CHIK^+^ patients, and not in DENV-2^+^ patients.

### Specific association of inhibitory KIRs on NK cells in DENV-2^+^ patients

**Fig 3:**
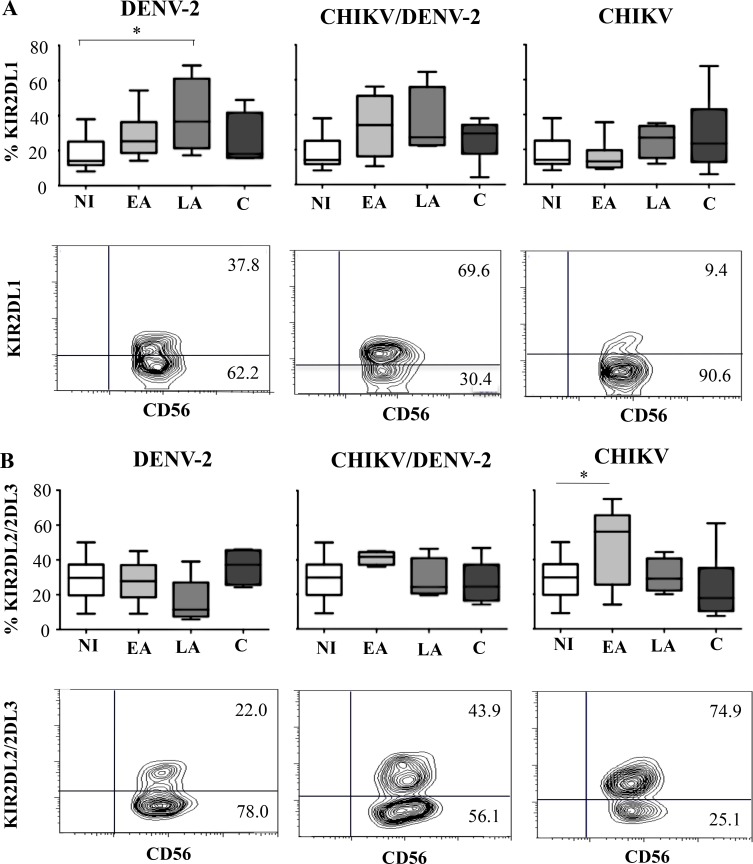
Association of KIR2DL1 expression and DENV-2 infection. Results are grouped according to type of infection: DENV-2, CHIKV or co-infected by CHIKV and DENV-2 (CHIKV/DENV-2), and compared to non-infected Gabonese healthy controls (NI). Samples from infected patients were collected in early acute (EA; day (D)0-D3), late acute (LA; D12-D15) and convalescent (C; D>30) stages post-onset of symptoms. Frequency of KIR2DL1 (A) and KIR2DL2/2DL3 (B) gated on CD3^-^CD56^+^ NK cells. Dot-plots show only cells from EA patients. * p<0.05, ** p<0.001.

KIR expression is a major event in the terminal differentiation of NK cells [22]. Fig 3 shows that in DENV-2-infected patients, the proportion of KIR2DL1^+^ NK cells was significantly increased (40±21% *vs* 17±9% in controls; p<0.05), whereas simultaneously the proportion of KIR2DL2/2DL3^+^ NK cells was decreased (16±12% *vs* 29±12% in controls). In contrast, in CHIKV^+^ patients, KIR2DL2/DL3 was significantly increased (48±15% *vs* 29±12% in controls; p<0.05) and KIR2DL1 decreased (14±2% *vs* 17±9%) (Fig 3), as previously described [14]. In co-infected patients, 35±19% of NK cells expressed KIR2DL1 (*vs* 17±9% in controls) and 41±4% KIR2DL2/2DL3 (*vs* 29±12% in controls) (Fig 3). These data suggest that DENV-2 and CHIKV induce two different profiles of NK cells.

**Fig 4:**
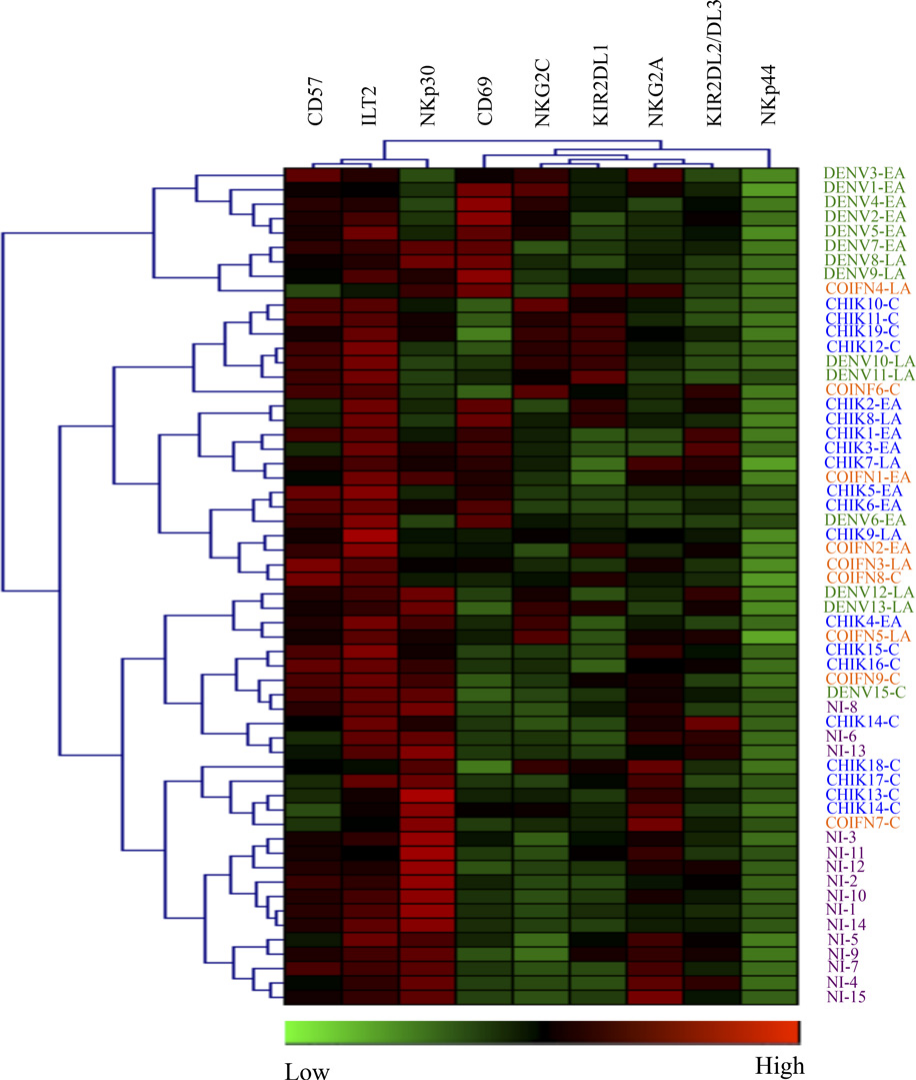
Hierarchical clustering analysis of 9 NK-cell markers from patients infected with DENV-2 (DENV; Green; n = 15), CHIKV (CHIK; Blue; n = 19), co-infected with CHIKV and DENV-2 (COIFN; Orange; n = 9), and non-infected healthy Gabonese controls (NI; Purple; N = 15). Patients were sampled in early acute (EA; day (D)0-D3), late acute (LA; D12-D15) and convalescent (C; D>30) stages post-onset of symptoms. Hierarchical clustering analysis was performed with the Genesis program (software available at www.genome.tugraz.at), as previously described [14,25]. Each column is dedicated to a definite NK marker. The color of each square reflects the percentage of expression of the corresponding marker in each individual. The values measured for samples were color displayed and rank ordered considering the healthy donors’ median as a reference: green color indicates inferior to median, and red color indicates superior to median, with values ranging from -3 to +3.

A hierarchical clustering analysis of nine surface markers was performed on CD3^-^CD56^+^ NK cells, as described [25]. Fig 4 shows that infected samples are easily distinguished from healthy control donors, whatever the time-point in the kinetics of the infection. Interestingly, at the early-acute phase of infection, NK cells derived from DENV-2-infected patients constitute an independent cluster, whereas CHIKV and co-infected samples tend to be mixed (Fig 4).

**Fig 5:**
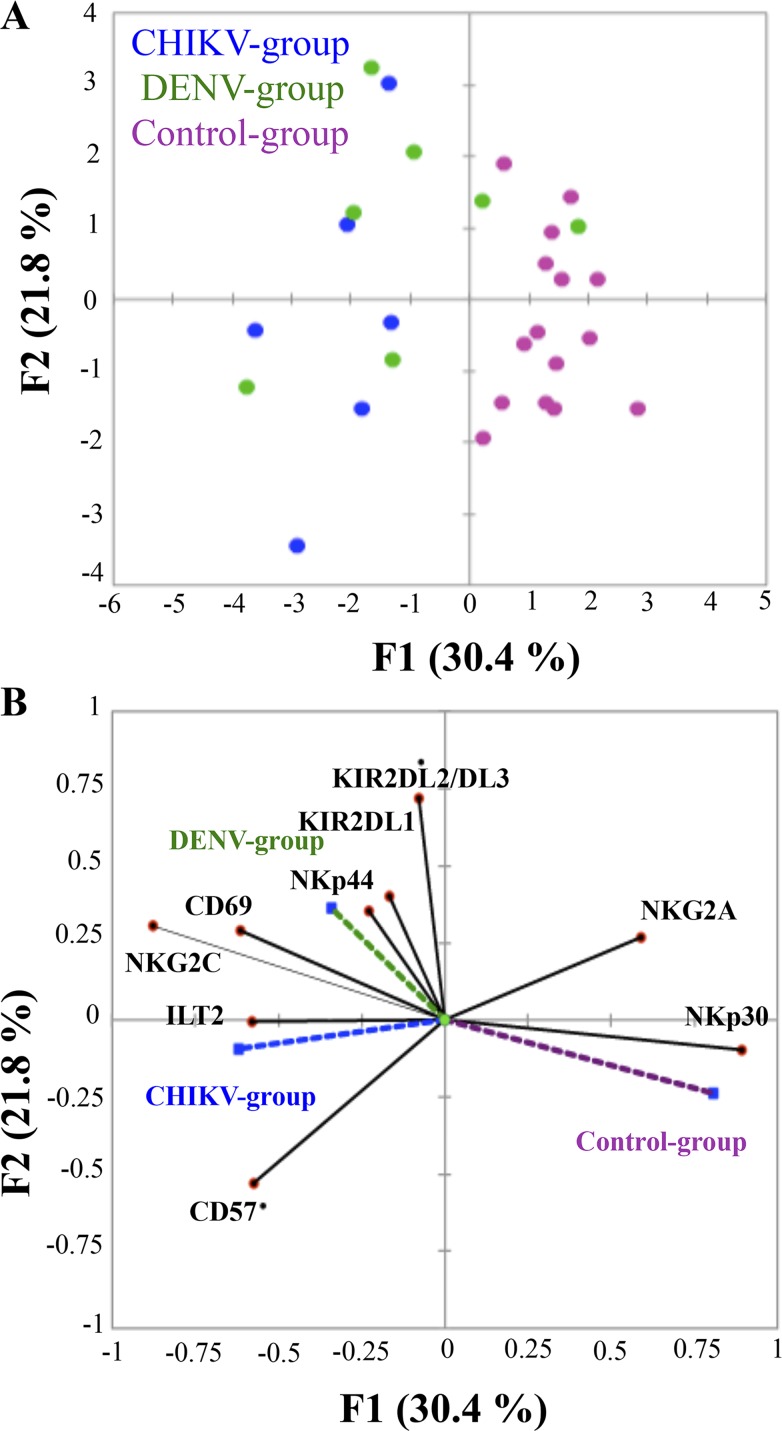
**Principal component analysis graphically showing the (A) statistical distribution of the individuals, according to the differential expression of NK-cell markers, and (B) statistical proximity between these different markers.** DENV-2^+^ patients are noted in green, CHIKV^+^ patients in blue, and healthy donors in purple.

To further confirm these observations we performed a principal component analysis (PCA) based on the expression of the different NK cells markers. Fig 5A shows that DENV-2-infected patients segregated distinctly from CHIKV-infected patients. NKp44, KIR2DL1 and KIR2DL2/3 markers were linked to the DENV-2^+^ patient group, whereas CD57 and ILT-2 markers mostly associated with CHIKV-infected patients. CD69 and NKG2C seemed to correlate with patients infected with either CHIKV or DENV-2 (Fig 5B). This combinatorial analysis unambiguously revealed that DENV-2 and CHIKV infections both induce cell-activation, but also two different signatures of NK-cell responses; CHIKV infection is associated with the terminal NK-cell differentiation (CD57), whereas, DENV-2 infection is mostly associated with the modulation of inhibitory KIRs and NKp44, a unique NK marker only expressed on activated NK cells.

### Functional activity of NK cells in DENV-2^+^ patients

**Fig 6:**
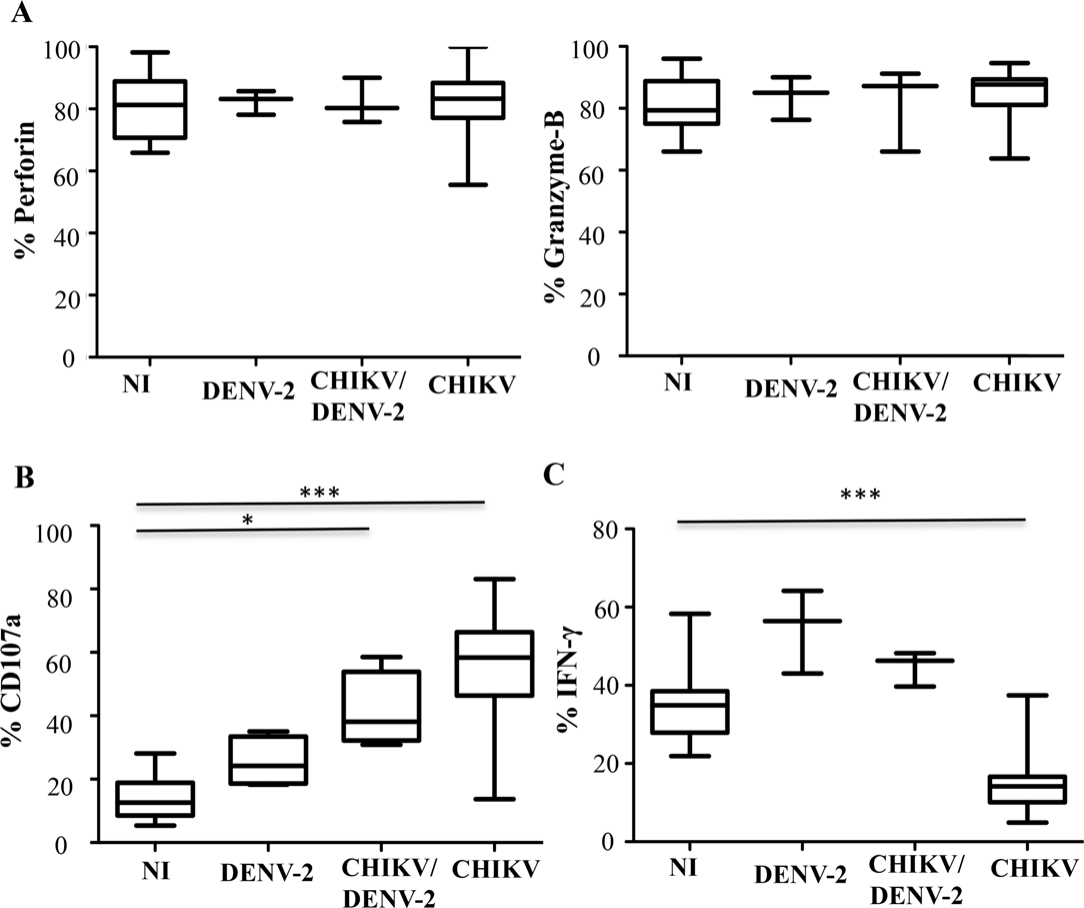
Cytolytic markers and functional activity of NK cells in patients infected with DENV-2, CHIKV, co-infected with CHIKV and DENV-2 (CHIKV/DENV-2), and non-infected and healthy Gabonese controls (NI). Patients were tested at early acute (EA; day (D)0-D3) stage post-onset of symptoms. (A) Intracellular staining of perforin and granzyme-B cytolytic markers on non-activated CD3^-^CD56^+^ NK cells. (B) CD107a expression on CD3^-^CD56^+^ NK cells stimulated by K562 target cells. Results are shown for an effector/target (E/T) cell ratio of 1/1. (C) Intracellular staining of IFN-γ^+^ in CD3^-^CD56^+^ NK cells after IL-12 plus IL-18 overnight stimulation. *: p<0.05; ***: p<0.0001.

We next assessed the overall functional capacity of NK cells during infection with DENV-2. NK cell cytolytic activity is mediated mainly by the release of lytic enzymes from cytotoxic granules and death receptor activation [26]. We determined the NK-intracellular levels of the lytic enzymes perforin, and granzyme-B in samples collected during the early-acute phase of infection by DENV-2 and/or CHIKV as well as in samples collected from healthy donors. Fig 6A shows that intracellular expression of perforin and granzyme-B was essentially equivalent in the various study groups. However, the NK cells capacity to release cytotoxic granules (degranulate), demonstrated by measuring the expression of CD107 after stimulating NK cells with HLA-class I negative K562 target cells, was increased in all infected patients and particularly in patients infected with CHIKV (Fig 6B), as previously described [14].

In addition to having a cytolytic function, NK cells play a central immunoregulatory role by selectively releasing various cytokines including IFN-γ. As previously described [14], the level of intracellular IFN-γ after treatment with IL-12 and IL-18 was significantly lower in CHIKV^+^ NK cells than in controls (Fig 6C). In contrast, NK cells from patients infected by DENV-2 or co-infected produced high levels of IFN-γ.

Together, these results suggest that NK cells display a polyfunctional profile very early after infection by DENV-2.

## Discussion

In this study, we investigated the immunological footprint of DENV-2 NK-cell responses, and we compared the phenotypic and functional characteristics of NK-cells during DENV-2, CHIKV and CHIKV/DENV-2 infections. Many studies have highlighted the importance of NK cells in controlling acute viral infections [27] yet the involvement of NK cells in response to mosquito-borne arboviruses is poorly appreciated [3,19]. Here we provide evidence that NK cells are involved in the earliest stages of the innate response to DENV-2, similarly to what was previously shown to occur during CHIKV infection [14]. It is important however to note the existence of significant differences in NK profiles between DENV^+^ and CHIKV^+^ patients on one hand for different NK cell markers but also regarding the modulation of NK cells subsets that occurs in a significantly slower manner in DENV-2 compared to CHIKV patients, in agreement with the kinetics of appearance of the first clinical symptoms of each infection. However, we observed a rapid activation of NK cells, previously described [17], in association with the accumulation of mature, terminally differentiated NK cells, expressing more frequently NKG2C and CD57 in both infections. A similar “clonal” expansion of CD57^+^NKG2C^+^ NK cells was previously reported in response to other infections, including HCMV, HIV-1, hantavirus, or viral hepatitis, [28–31], but also CHIKV [14]. There is growing evidence showing that this phenomenon only occurs in CMV seropositive patients [21]. In the present cohort and depending on the patient group, seroprevalence of HCMV ranged between 87.5 and 100%, which is similar to published data for the African population [32]. The expansion of NKG2C^+^ NK cells observed here early after DENV-2 infection could therefore also be linked to HCMV status. In DENV-2^+^ patients, the activation and expansion of NK cells was transient and values returned to baseline levels about one month after disease onset. In contrast, CHIKV^+^ patients presenting with the highest viral loads during the acute stage of infection also showed prolonged persistence of NKG2C^+^CD57^+^ NK cells. Given the fact that all these patients further developed chronic arthralgia, it is tempting to speculate that the acute stage of CHIKV infection may affect both the outcome of the disease and the development of persistent symptoms by acting to maintain a particular inflammatory environment. Several studies have also suggested a link between chronic disease and a stronger inflammatory response in the acute phase of infection [33,34]. However, further investigations are required to correlate these different features with disease progression.

Skewing of inhibitory KIR repertoire towards self-specific KIRs has previously been observed in virus-infected patients [30,35]. Here we show that CHIKV infection was associated with an increased expression of KIR2DL2/DL3, whereas, DENV-2 induced the expansion of KIR2DL1^+^ NK cells. We recently demonstrated that CHIKV infection was certainly associated with an interplay between KIR2DL1 and HLA-C2, whereas, DENV-2 infection seems to not act through a specific KIR/HLA pathway [36]. The mechanisms behind the expansion of NK cells bearing self-specific KIR remain elusive; we can only hypothesize that HLA-presented DENV-2 peptides could modulate KIR/HLA interaction as previously shown in HIV-1 infected patients [10,37]. Hence, certain peptides from DENV-2 may be preferentially associated with HLA-C2 molecules, which recognize KIR2DL1, in accordance with previous studies providing evidence that DENV evades NK triggering through MHC-I enhancement [38,39]. Further studies will be required to identify these HLA-restricted viral peptides and the functional consequences in regards to the NK cell response [40].

A hierarchical clustering analysis of a panel of phenotypic markers of the NK cell subset only strengthened the evidence of NKR repertoire modulation, showing that CHIKV- and DENV-2-infected patients develop different NK phenotypes, distinct from those of Gabonese healthy donors, and that NK cells from early-acute DENV-2^+^ patients constitute a specific cluster. Principal component analysis highlighted a privileged association of NKp44 with DENV-2 infection. It is important to note that Hershkovitz et al [41] have previously reported a direct interaction between NKp44 and the envelope (E) protein of DENV, but also of West Nile virus, leading cause of meningitis-encephalitis, suggesting a common and specific role of NKp44 in flavivirus infections. We can however speculate that the NKp44-mediated outcome of the interaction between NK cell and flavivirus-infected cell is affected by the expression of its cellular ligand. Recently, we demonstrated that NKp44 triggers NK-cell cytotoxicity upon recognition of a new Mixed Lineage Leukemia 5 (MLL5) isoform, called NKp44L, expressed only on certain stressed cells [42]. Nonetheless, the expression of NKp44L on DENV-target cells remains to be explored to comprehensively enlighten the physiological relevance of NKp44 engagement in NK cells from DENV-infected patients.

DENV circulates in regions that are prone to many other endemic diseases, such as CHIKV, West Nile virus, yellow fever, malaria, and more recently Zika virus, for which *Aedes* species mosquitoes are also potent vectors [43–45]. Little to nothing is known in regard to the host’s immune responses or to the subsequent clinical manifestations and outcome of patients co-infected by vector-borne pathogens. To the best of our knowledge, this is also the first study to explore the NK cell phenotype of CHIKV/DENV-2 co-infected patients. Caron *et al* [4] have shown that co-infected patients can be subdivided according to their respective CHIKV and DENV-2 viral load levels, suggesting a possible mechanism of viral competition. Within this complex scenario, our data reveal that the NK-cell response triggered by CHIKV/DENV-2 co-infection reflects a combination of the results observed in mono-infected patients, in terms of potency and durability (ie NKp30, NKG2A and CD161), and a unique profile of KIR2DL2/2DL3 and KIR2DL1 co-expression.

NK cells of patients infected by CHIKV and/or DENV-2 appeared to be highly cytotoxic at the peak of infection, particularly in DENV-infected patients, as was previously shown [14]. Importantly, contrasting with CHIKV-infected patients, NK cells from DENV-2^+^ samples present an increased capacity to produce IFN-γ during the acute phase of infection. Consistently with these data, increased quantities of plasmatic IFN-γ have been reported early after the one-set of the symptoms in DENV-2-infected patients [46,47]. Clearly, additional longitudinal functional analyses are required to determine whether NK-cell functions are sustained beyond the first few days of DENV infection.

We hypothesize that NK cells are strongly involved during acute DENV-2 infection. Improving our understanding of the immune mechanisms that control arboviral infections is crucial in the current race against the globalization of these epidemics. The emergence of co-infections and the unprecedented increase in magnitude in morbidity and mortality during recent major concomitant outbreaks are concerning new threats which need to be closely monitored.

## Supporting Information

S1 Fig Frequencies of HLA-DR, NKp44 and ILT2 within CD3^-^CD56^+^ NK cells collected from patients infected by DENV-2, CHIKV, co-infected by CHIKV and DENV-2 (CHIKV/DENV-2), and non-infected Gabonese healthy controls (NI). Samples were collected in early acute (EA; day (D)0-D3), late acute (LA; D12-D15) and convalescent (C; D>30) stages post-onset of symptoms.
(TIF)
