# Supplementary figures and images for: Longitudinal Analysis of Natural Killer Cells in Dengue Virus-Infected Patients in Comparison to Chikungunya and Chikungunya/Dengue Virus-Infected Patients
Source: PLoS Negl Trop Dis. 2016 Mar 3;10(3):e0004499. doi: 10.1371/journal.pntd.0004499 (PMC4777550; doi:10.1371/journal.pntd.0004499)

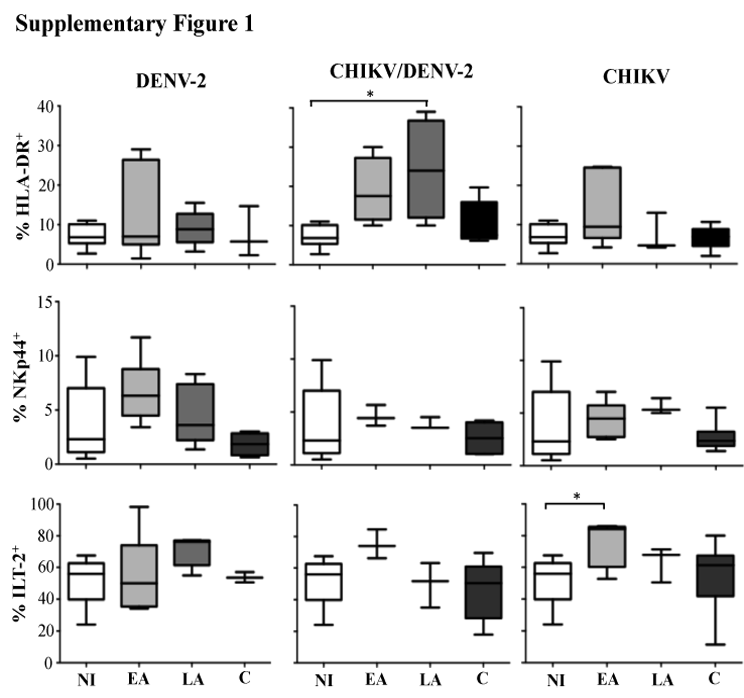

Supplement: S1 Fig — Samples were collected in early acute (EA; day (D)0-D3), late acute (LA; D12-D15) and convalescent (C; D>30) stages post-onset of symptoms. (TIF) [file pntd.0004499.s001.tif]
